# Components of the Endocannabinoid System and Effects of Cannabinoids Against Bone Diseases: A Mini-Review

**DOI:** 10.3389/fphar.2021.793750

**Published:** 2022-01-19

**Authors:** Yuqi Xin, Anqun Tang, Shuting Pan, Jie Zhang

**Affiliations:** Department of Oral and Maxillofacial Surgery, The First Affiliated Hospital of Nanchang University, Nanchang, China

**Keywords:** cannabinoids, bone loss, osteoporosis, osteoarthritis, bone tumor, bone fractures

## Abstract

**Background:** The endocannabinoid system (ECS) is involved in multiple physiological processes, including appetite regulation, pain perception, motor function development, and immune response regulation. Cannabinoids have been approved for the clinical treatment of nausea and vomiting caused by cytostatic therapy or cancer chemotherapy, loss of appetite in HIV/AIDS-associated cachexia, refractory spasms induced by multiple sclerosis, chronic pain, and urinary incontinence.

**Methods:** Check out the research on ECS and bone diseases in the past 20 years.

**Results:** Many studies have demonstrated that endocannabinoids (eCBs) and cannabinoid receptors (CBRs) are expressed in bone and synovial tissues, playing important roles in bone metabolism. Preclinical studies using cannabis-based therapies in animal models have shown that cannabinoids (CBs) can alleviate the development of osteoarthritis (OA), prevent osteoporosis (OP), reduce cancer-induced osteolytic destruction, and improve fracture healing, highlighting the therapeutic potential of CBs for human bone diseases.

**Conclusions:** The present review summarizes various components of the ECS in bone diseases and their potential as a therapeutic target.

## Introduction

Cannabis Sativa has been used medicinally and recreationally for thousands of years. As early as in 2600 B.C., cannabis was already used in treating malaria, constipation, pain and dysmenorrhoea in China (Mechoulam, 1986; Grinspoon, 1993). In the late 19th century, European people began using cannabis to treat pain, muscle spasms, asthma, insomnia, depression and anorexia. However, it was not until 1964 that its major active chemical component delta-9-tetrahydrocannabinol (THC), also known as dronabinol, was discovered (Gaoni and Mechoulam, 1964). Nearly 30 years later, a specific cannabinoid receptor (CBR) was identified as the target of THC (Devane et al., 1988; Munro et al., 1993). In 1992, Devane et al. discovered the first endocannabinoid (eCB) N-arachidonoylethanolamine or anandamide (AEA) as the endogenous ligand of CBR in the pig brain (Devane et al., 1992)^.^ Subsequently in 1995, the second eCB 2-arachidonoylglycerol (2-AG) was also discovered (Mechoulam et al., 1995). With the discovery of endocannabinoids, a great number of studies have investigated the physiological functions of the endocannabinoid system (ECS).

The ECS is recognized to play a significant role in regulating a variety of physiological processes, including appetite control, pain perception, and immune regulation (Idris and Ralston, 2010; Robson, 2014). Marinol (dronabinol), a cannabinoid receptor 1(CB1) agonist, has been approved in the United States of America (USA) for the clinical treatment of nausea and vomiting, and anorexia caused by cytostasis or AIDS. Nabilone has also been approved in the United Kingdom (UK) for the treatment of chemotherapy-induced adverse effects in cancer patients. A new CB drug (sativex) has also be approved in Germany for the treatment of intractable muscle spasm caused by multiple sclerosis (Grotenhermen and Müller-Vahl, 2012).

Several research groups have reported ECS expression in bone and synovial tissues and its important role in bone metabolism (Carnovali et al., 2016; Ehrenkranz and Levine, 2019; Dou et al., 2020). Preclinical studies in animal models demonstrated that CBs could alleviate the development of arthritis, prevent osteoporosis (OP), inhibit bone tumor cell proliferation, reduce bone cancer pain and improve fracture healing (Smoum et al., 2015; Carnovali et al., 2016; Frei et al., 2016; Marino et al., 2019; Yan et al., 2019). In this regard, recent progress in the application of CBs in bone diseases has been reviewed, with the expectation to provide a new direction for the clinical treatment of bone diseases. In this article, we will discuss the potential therapeutic effects of CBs for the treatment of patients with bone diseases.

## CB Expression in Bone and Joint Tissues

The ECS consist of endogenous cannabinoid ligands (endocannabinoids, eCBs), their receptors, and the gene enzymes involved in their synthesis and degradation (McPartland et al., 2006; Pertwee et al., 2010). Arachidonoylethanolamine (AEA) and 2-Arachidonoylglycerol (2-AG) are two firstly identified and most studied eCBs, which are believed to be involved in a wide range of physiological processes including appetite stimulation, pain modulation and energy expenditure (Di Marzo and Petrosino, 2007). Cannabinoid receptor 1 (CB1) and 2 (CB2) are the characterised cannabinoid receptors, to which AEA and 2-AG bind in the nanomolar range. 2-AG is considered a full agonist, and AEA is considered a partial agonist (Pertwee and Ross, 2002). Other receptors known for eCBs include G-protein coupled receptors (GPR55 and GPR119), transient receptor potential vanilloids (TRPV1 and TRPV4), peroxisome proliferator-activated receptors (PPARα and PPARγ), and various ion channels (Pertwee, 2010).

The ECS exists in most mammalian organs and tissues, playing primarily important roles in the nervous and immune systems (Joshi and Onaivi, 2019; Silver Robert, 2019). ECBs and their receptors are also expressed in the bone (Rossi et al., 2009; Whyte et al., 2009). CB1, CB2 and TRPV1 have been identified in human osteoclasts (OCs) and GPR55, and found to be expressed in both human osteoblasts (OBs) and OCs (Rossi et al., 2009; Whyte et al., 2009). Mouse OBs and OCs express CB1, CB2, GPR55 and TRPV1 (Idris et al., 2005; Ofek et al., 2006; Idris et al., 2009; Whyte et al., 2009; Idris et al., 2010). Studies of the innervation of the mouse bone have shown that CB1 and TRPV1 are expressed in sympathetic nerve fibers (Ghilardi et al., 2005; Tam et al., 2006). AEA and 2-AG are responsible for most of the pharmacological effects associated with CBRs in mammalian cells (Pertwee, 2005). Jiang S et al. discovered AEA and 2-AG are produced in bone marrow (Bab et al., 2008; Tam et al., 2008; Jiang et al., 2010). AEA and 2-AG are detectable in human OC- and OB-like cells *in vitro* (Rossi et al., 2009; Whyte et al., 2012). Rossi et al. (2009) reported that cultured human OCs produced 2-AG and a certain amount of AEA *in vitro*, and the level of eCBs increased when the culture was treated with URB597, a fatty acid amide hydrolase (FAAH) inhibitor. Whyte et al. (2012) reported that differentiation of human OCs was related to the increased AEA level and decreased 2-AG level. These observations suggest that AEA and 2-AG may be produced by bone cells and within the cultured bone (Tam et al., 2008; Maccarrone et al., 2015). The enzyme diacylglycerol lipases *a* and *ß* and NAPE-phospholipase D, which are required for 2-AG and AEA synthesis respectively, are also expressed in OBs and OCs (Tam et al., 2008; Rossi et al., 2009). Similarly, the degradation enzymes FAAH and monoacylglycerol lipase (MAGL) have been found in human OCs and mouse OBs (Hutchins et al., 2011; Rossi et al., 2011). The role of cannabinoid receptor ligands in regulating osteoclasts, osteoblasts and adipocytes *in vitro* and *in vivo* are shown in Table 1.

**TABLE 1:** The role of cannabinoid receptor ligands in regulating osteoclasts, osteoblasts, and adipocytes *in vitro* and *in vivo*.

	Ligand	Receptor	Bone metabolism			
			Oc	Oc	Ob	Ac
			Number	Activity	Number	Number
Agonists	AEA	CB1/CB2/GPR55/TRPV1	↑	↑	↑	-
	2-AG	CB1/CB2/GPR55	↑	↑	↑	-
	Δ9-THC	CB1/CB2	-	-	-	-
	CP55,940	CB1/CB2	↑↓	↑	↑	↓
	WIN55,212	CB1	-	-	↑	-
	HU308	CB2	↑↓↓	-	↑↑	↓
	JWH133	CB2	↑	↑	↑	↓
	JWH139	CB2	-	-	-	-
	JWH015	CB2	-	↑	↑	↓
	AM1241	CB2	-	-	-	-
	Lysophosphatidyl inositol	GPR55	↓	↑	-	-
	O-1602	GPR55	↓	↑	↑	-
Antagonists	AM630	CB2>CB1/GPR55	↓↓↑	↓	↓	-
	SR144528	CB2>CB1	↓	↓	↓	-
	AM251	CB1>CB2/GPR55	↓↓↑	↓	↓	↑
	SR141716A	CB1>CB2	↓↓	↓	↓	-
	Cannabidiol	GPR55	↑	↓↓	-	-

Abbreviations: CB1, cannabinoid type 1 receptor; CB2, cannabinoid type 2 receptor; GPR55, G protein-coupled receptor 55; TRPV1, transient receptor potential vanilloid type 1. Oc., osteoclast; Ob., osteolblast; Ac., adipocyte. ↑, increase; ↓, decrease; –, non tested. Black and red arrows denote *in vitro* and *in vivo* data, respectively.

The ECS is also expressed in synovial tissues of joints. Richardson et al. (2008) reported that CB1 and CB2 receptors were expressed in synovial biopsies of human osteoarthritis (OA) and rheumatoid arthritis (RA) by Western Blot detection, and played a role in regulating physiological functions. Further evidence showed that AEA and 2-AG could be detected in synovial fluid from OA and RA patients but not in synovial fluid from normal subjects. Dunn et al. (2016) reported that a wide range of CBRs including CB1, CB2, GPR55, PPARαand PPARγ were expressed in chondrocytes of OA joints, and even in degenerate tissues.

## Cannabinoids Affect Bone Diseases

### Osteoporosis

OBs are known to synthesize bone cells to produce AEA and 2-AG, and express CB1 receptors on their surfaces (Tam et al., 2008; Maccarrone et al., 2015). Activation of CB1 in OBs inhibits the release of norepinephrine, which to some extent suppresses the process of bone formation, i.e., CB1 activation inhibits bone production (Tam et al., 2006). In addition, OCs also express AEA and 2-AG, but with CB2 receptors instead of CB1 (Whyte et al., 2012). CB2 activation in OCs suppresses osteolysis activity, thereby preserving the bone tissue (Whyte et al., 2012). This effect proves highly beneficial to balancing the relationship between hyperactive OCs and inactive OBs in OP, leading to increased bone resorption without compensatory bone formation. These findings support that the ECS is the main regulatory system of the bone. Although norepinephrine is directly responsible for the activities of OCs and OBs, the level of norepinephrine is mainly mediated both by the ECS expressed in the sympathetic nervous system and that expressed in the bone tissue itself.

CBs have been shown to regulate bone formation, bone loss and bone turnover. The ECS system is an important regulator of bone mass. CBR agonists promote differentiation of mouse mesenchymal stem cells (MSCs) into OBs (Zhang et al., 2019). Idris et al. (Hutchins et al., 2011) reported that CB1 receptor inactivation increased bone mass and prevented bone loss due to ovariectomy, an *in vivo* model of OP in 2005. In addition, Rossi et al. reported that CB2 receptors had an anti-osteoporosis function (Rossi et al., 2019). CB2 receptor agonists increased bone mass by enhancing the number and activity of OBs, inhibiting the proliferation of OCs, and stimulating fibroblastic colony formation by myeloid cells (Ofek et al., 2006). Furthermore, CB2 receptor regulates bone loss also involving the regulation of osteoclast function (Sophocleous et al., 2017). Therefore, CB2 provides a molecular target for the diagnosis and treatment of OP.

GPR55 was expressed in human and mouse OCs and OBs. In contrast to the bone turnover function of CB1 and CB2 receptors, GPR55 inhibited OC formation but stimulates OB function. Histomorphometric and microcomputed tomography analysis of the long bones in male GPR55 (−/−) mice revealed that the number of OCs was increased, but the volume and thickness of the trabecular bone was increased significantly with no cartilage resorption observed, a possibility is that osteoclast numbers were increased, but osteoclast function was impaired (Whyte et al., 2009). Therefore, GPR55 receptor agonists promote bone loss. A recent study of OCs from patients with OP suggested that GPR55 desensitization by FAAs or its enhanced transport, and TRPV1 agonist-induced overexpression of CB2 receptor might be critical to reduce calciumentry into OCs, which could lead to over-activation of cells and increase bone resorption and bone loss. TRPV1 agonistsCB together with CB2 agonists were reported useful for the treatment of OP (Rossi et al., 2011). These results indicate that CBRs agonists could be used for the prevention and treatment of OP.

### Osteoarthritis

OA is characterized by degeneration of the articular cartilage, which is mediated by complex interactions of proinflammatory cytokines including IL-1, inflammatory mediators and proteases. CBs have been shown to prevent IL-1-induced matrix breakdown of collagen and proteoglycan, suggesting that they may play an important role in cartilage protection (Mbvundula et al., 2006). CBs exert their effects through several CBRs, and therefore it is important to identify the key CBs and CBRs involved in cartilage protection. CBRs are expressed in synovial tissues and osteoarthritic articular chondrocytes and produce important physiological effects such as reducing arthritis inflammation and alleviating arthritis-associated pain symptoms (Mbvundula et al., 2006; Guidetti et al., 2014). Malfait et al. (2000) described the effect of a high potency dimeric cannabinoid, named cannabisol (CBD), in a mouse arthritis model and found that CBD had immunosuppressive and anti-inflammatory activities and could improve the symptoms of arthritis in a murine collagen-induced arthritis model by both intraperitoneal and oral administration methods. They also found that CBD could reduce joint damage. The *in vitro* effects of CBD included dose-dependent inhibition of lymphocyte proliferation, both mitogen-stimulated and antigen-specific, and reactive oxygen burst triggered by peritoneal granulocytes blocking zymosan. It was also found that CBD administration could block the increase of serum tumor necrosis factor (TNF) induced by lipopolysaccharide in C57/BL mice. Sumariwalla et al. (2004) used a synthetic CB (HU-320) in a similar experiment and found that this novel synthetic CB HU-320 could be used to treat arthritis in mice due to its strong anti-inflammatory and immunosuppressive properties without showing psychoactive effects. HU-320 inhibited the production of tumor necrosis factor (TNF) and reactive oxygen intermediates (ROIs) from mouse macrophages and RAW 264.7 cells respectively, as well as the increased serum TNF level following endotoxin attack.

Oversecretion of proinflammatory cytokines from OBs plays an essential role in the development of OA (Liu et al., 2014; Sun et al., 2014), and high levels of pro-inflammatory factors in bones and joints induce pain, cartilage loss, and even joint dysfunction (Yang et al., 2013; Karsdal et al., 2014). Therefore, reducing the release of pro-inflammatory cytokines from OBs is an effective therapy for OA. Yang et al. (2015) have reported that THC inhibited the release of pro-inflammatory cytokines, including TNF-α, IL-1, IL-6, and IL-8, decreased nuclear factor-B (NF-B) expression, and inhibited the upregulation of cofilin-1 protein (a cytoskeleton protein involved in inflammation of OA of lipopolysaccharide (LPS)-stimulated MG-63 cells. The administration of the CB2 receptor antagonist or the CB2-siRNA partially abolished the above-mentioned THC-induced anti-inflammatory effect. In addition, overexpression of cofilin-1 significantly reversed the THC-induced anti-inflammatory effect in MG-63 cells. These results indicate that CB2 is involved in the anti-inflammation induced by THC in LPS-stimulated MG-63 cells, and suggest that the anti-inflammatory effect may be mediated by cofilin-1 (Yang et al., 2015).

Cannabinoids exert chondroprotective effects and are useful for OA treatment (Dunn et al., 2012). Dunn et al. (Dunn et al., 2016) designed CBs to bind to their receptors and found that they inhibited the catabolic and pain pathways within the arthritic joint without causing psychoactive effects, suggesting a therapeutic potential for arthritis. Sophocleous et al. (2015) reported that CB2-selective agonist HU308 reduced the severity of total knee joint OA following surgical destabilization of the medial meniscus (DMM) in wide type mice. When compared with wild-type chondrocytes, cultured articular chondrocytes from CB2 deletion (CB2^−/−^) mice produced less proteoglycans *in vitro*, indicated that the CB2 pathway played a role in the pathophysiology of murine OA, and that the pharmacological activity of CB2 had a protective effect against OA.

There is increasing evidence that the ECS, especially CB2, also plays an important role in the pathophysiology of rheumatoid arthritis (RA) (Lowin et al., 2019). Gui et al. (2014) reported that many members of the ECS inhibit synovial inflammation, hyperplasia, and cartilage destruction in RA. In particular, activation of CB2 may relieve RA by inhibiting the production of autoantibodies, proinflammatory cytokines, and matrix metalloproteinases (MMPs), as well as bone erosion, T cells mediated immune response, and the proliferation of FLSs (Gui et al., 2014).

CBs can also reduce the loss of thealveolar bone (Ossola et al., 2016). Ossola et al. have demonstrated that CB-2 receptor was expressed in OBs and OCs to promote bone metabolism. And the results of their studies in rat models showed that alveolar bone loss was greatly attenuated by the use of CB-2 receptor agonist HU-308 in LPS-induced periodontitis and as such demonstrated anti-inflammatroy, osteoprotective, prohomeostatic effects (Ossola et al., 2016).

CBs can not only reduce the inflammation of arthritis but relieve the pain symptoms of arthritis. The termination of endocannabinoid activity is achieved by cellular uptake, followed by intracellular hydrolysis by fatty acid amide hydrolase (FAAH) (Schuelert et al., 2011). Schuelert et al. (2011) indicated local injection of FAAH inhibitor URB597 into the OA knee joints reduced mechanical nociception and pain in two OA rodent models, and this response was eliminated by CB1 receptor antagonists, indicating that CB1 receptor could be used as an arthritic pain treatment target.

### Bone Tumors

Several studies have demonstrated the positive effects of the ECS in the treatment of cancer. CBRs can inhibit tumor cell proliferation, reduce tumor cell invasion, cause tumor regression and prevent tumor metastasis. For example, Qamri et al. (2009) reported that synthetic CBR agonists inhibited tumor growth and metastasis of breast cancer. Preet et al. (2008) indicated that delta9-Tetrahydrocannabinol inhibited lung cancer cell migration *in vitro* as well as its growth and metastasis *in vivo*. Gustafsson et al. (2008) showed that therapeutic options using ABR ligands had the efficacy of reducing tumor burden in malignant lymphoma overexpressing CB1 and CB2. However, the role of CBs in bone metastasis remains to be studied.

There have been many studies concerning the use of the ECS in the treatment of primary bone tumors. For tumor cells, ECS can affect their growth, movement, invasion, spread, and colonization of distant organs. It was found that CB2 was modulated in the genetic and phrenological processes, thus affecting bone cell activity in remodeling in both healthy individuals and patients (Di Marzo, 2008; Marino and Idris, 2017). Furthermore, Niu et al. (2015) have demonstrated that the synthetic cannabinoid CB receptor agonist WIN-55,212-2 has therapeutic effect on the MG-63 human osteosarcoma cell line. WIN-55,212-2 inhibit migration, invasion and angiogenic activity of this cell line. The mechanism of this inhibition is associated with the downregulation of the Notch-1 and MMP-2 signaling pathways, which are known as important pathways associated with cell proliferation and apotosis as well as degradation of extra-cellular matrix, the key to tumor invasion. High level of MMP-2 is considered a key indicator of cancer metastasis (Berx et al., 1998; Jezierska and Motyl, 2009; Niu et al., 2015).

Lozano‐Ondoua et al. noted that the CB2 receptor agonist reduced the degree of tumor burden within the intramedullary cavity of the femoris and produced anti-progressive effects of the tumor *in vivo* (Lozano-Ondoua et al., 2013).

Lozano-Ondoua et al. (2010) showed that CB2 receptor agonists could attenuate sarcoma-induced pain, reduce cancer-induced osteolytic destruction, and prevent the occurrence of pathological bone fracture. CB1 and CB2 were found to be associated with mediating ligands and molecular mechanisms associated with synthesis, transport and metabolism with potential effects of reducing complications of primary bone tumors. Especially, CB1 and CB2 agonists reduced bone cancer pain in animal models (Curto-Reyes et al., 2010; Kawamata et al., 2010). Therefore, this approach may be applied as analgesic treatment in patients with Bone tumors. The role of cannabinoid receptor ligands in regulating tumor cells and bone diseases *in vitro* and *in vivo* are shown in Table 2.

**TABLE 2:** The role of cannabinoid receptor ligands in regulating tumor cells and bone diseases *in vitro* and *in vivo*.

	Ligand	Receptor	Bone tumor		
			Tm.Growth	Osteolysis	Pain
Agonists	AEA	CB1/CB2/GPR55/TRPV1	↓↓	-	↓
	2-AG	CB1/CB2/GPR55	↓	↓	-
	Δ9-THC	CB1/CB2	↓↓↑↑	-	↓
	CP55,940	CB1/CB2	↓	-	
	WIN55,212	CB1	↓↓	-	
	HU308	CB2	↑↓	↑	-
	JWH133	CB2	↑↓↓	↓↓↑	
	JWH139	CB2	↓	-	
	JWH015	CB2	↓↓	↓	↓
	AM1241	CB2	↓	↓	
	Lysophosphatidyl inositol	GPR55	-	-	-
	O-1602	GPR55	-	-	
Antagonists	AM630	CB2>CB1/GPR55	-	↓	
	SR144528	CB2>CB1	-	-	
	AM251	CB1>CB2/GPR55	-	-	
	SR141716A	CB1>CB2	-	-	
	Cannabidiol	GPR55	↓↓	-	↓

Abbreviations: CB1, cannabinoid type 1 receptor; CB2, cannabinoid type 2 receptor; GPR55, G protein-coupled receptor 55; TRPV1, transient receptor potential vanilloid type 1. Tm., tumor cell. ↑, increase; ↓, decrease; –, non tested. Black and red arrows denote *in vitro* and *in vivo* data, respectively.

### Bone Fractures

Bone fractures are highly prevalent, involving prolonged immobilization and discomfort. Some researchers have found that CBRs trigger bone formation and strengthen the bridge that connects broken bones (Kogan et al., 2015). Kogan et al. (2015) reported that the major non-psychoactive cannabis constituent CBD enhanced the biomechanical properties of rat mid-femoral fracture healing. Micro–computed tomography (μCT) showed that the fracture callus size was transiently reduced by either CBD or THC 4 weeks after fracture but reached control level after 6 and 8 weeks. The callus material density was unaffected by CBD and/or THC. In contrast, CBD stimulated mRNA expression of Plod1 in primary OB cultures to encode an enzyme that catalyzes lysine hydroxylation, which in turn was involved in collagen cross-linking and stabilization. These data show that CBD can improve fracture healing and plays a critical mechanical role of collagen cross-linked enzymes. The bones of rats treated with CBD alone not only healed faster but the previous fracture was less likely to break in the future because of a strengthened fracture callus. Therefore, CBD provides research directions for the treatment and prognosis of fractures.

Bab et al. (2011) showed that fatty acid amides (FAAs) assisted in the process of bone metabolism by interacting with CBRs. FAAs are important because they are broken down by a particular enzyme (FAAH) that is blocked by CBD. For quite some time, CBD was known to inhibit FAAH, knowing that it could prevent the enzyme from breaking down bone forming compounds. An effective function of bone anabolic-antiresorptive is shared by many skeletal FAAs. Inhibition of the FAA degrading enzyme (FAAH) may prove to be an effective therapeutic strategy for the treatment of bone fractures.

**FIGURE 1:**
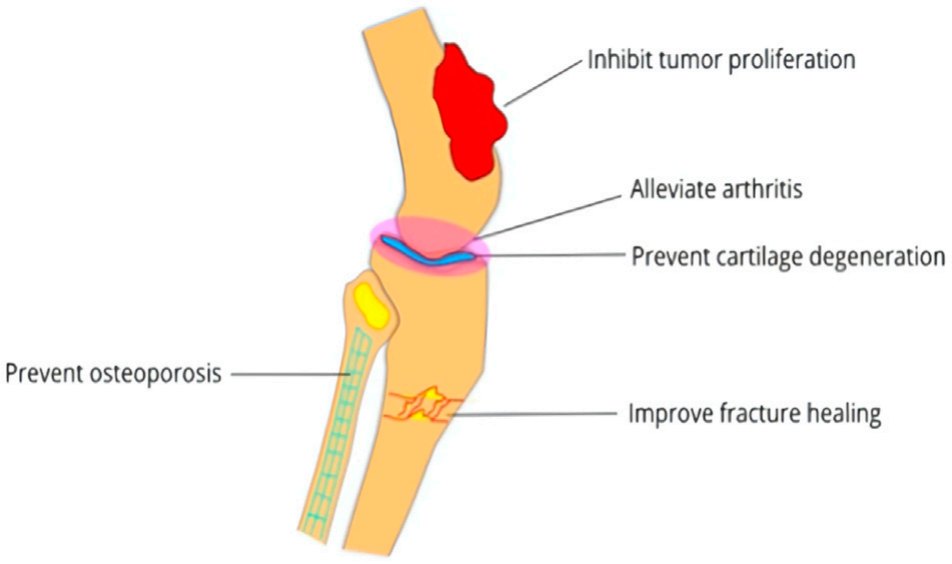
The potential therapeutic effects of cannabinoids in bone diseases.

In summary, CBs could alleviate the development of arthritis, prevent osteoporosis, inhibit bone tumor cell proliferation, reduce bone cancer pain and improve fracture healing (Figure 1).

## Conclusion

Bones provide structural support and physical protection to our soft tissues and allow us to walk, eat, breed and carry out the life activities. The ECS has been shown to regulate bone metabolism. An eCB deficiency may affect the skeletal system. No CB compound has been approved for the treatment of bone diseases at present. However, the important skeletal actions of the ECS have prompted both preclinical researchers and industrial companies to explore the clinical therapeutic potential based on CBs in the treatment of various bone diseases.

Evidence indicates the potential role of the ECS in the treatment of bone diseases because of the multiple targets involved in the pathologic process of various bone conditions including OP, OA, bone tumors and bone fractures. However, there is currently limited research on the use of CBs in the treatment of bone diseases, and the evaluation of medicinal cannabis in humans remains in its infancy. The following work still need to be further explored.

Firstly, the effect of CBR regulation on bone tissue metabolism needs further investigation in details. As it is necessary to further study the regulatory mechanisms of eCBs on osteogenesis, bone loss, synovial inflammatory response and arthritis pain, comprehensive evaluations of *in vitro* and *in vivo* mechanisms and pharmacologicals should be performed on each member of the eCB family. Secondly, to ensure clinical applicability of CBs, it is necessary to explore new ways to improve the therapeutic effects of CBs and reduce their neurological adverse effects, such as synthesizing new CB drugs and using CB hydrolase inhibitors to increase endogenous levels of CBs. Finally, further investigations on the function of the ECS and its role in bone diseases are required to provide a solid foundation for the evolution and refinement of cannabis-based medicines. Comprehensive evaluations through high-quality randomized controlled trials (RCTs) are also required to identify the true clinical efficacy and long-term risks associated with CB therapy.

The ECS plays a role in maintaining the bones strength and combating bone diseases, and holds promise as a novel drug for bone disease treatment.

## Abbreviations

ECS, The endocannabinoid system; eCB, endocannabinoid; CBR, cannabinoid receptor; CB, cannabinoid; OA, osteoarthritis; OP, osteoporosis; THC, delta-9-tetrahydrocannabinol; AEA, anandamide; 2-AG, 2-arachidonoylglycerol; OP, osteoporosis; GPR, G-protein coupled receptors; TRPV, transient receptor potential vanilloids; PPAR, peroxisome proliferator-activated receptor; OCs, osteoclasts; OB, osteoblasts; FAAH, fatty acid amide hydrolase; MAGL, monoacylglycerol lipase; CBD, cannabisol; TNF, tumor necrosis factor; NF-B, nuclear factor-B; LPS, lipopolysaccharide; MMPs, matrix metalloproteinases; RA, rheumatoid arthritis.
